# Stress and work-related mental illness among working adults with ADHD: a qualitative study

**DOI:** 10.1186/s12888-022-04409-w

**Published:** 2022-11-30

**Authors:** Martin Oscarsson, Martina Nelson, Alexander Rozental, Ylva Ginsberg, Per Carlbring, Fredrik Jönsson

**Affiliations:** 1 Department of Psychology, Stockholm University, 106 91 Stockholm, Sweden; 2 Department of Psychology, Uppsala University, Uppsala, Sweden; 3 Centre for Psychiatry Research, Department of Clinical Neuroscience, Karolinska Institutet, & Stockholm Health Care Services, Stockholm, Sweden

**Keywords:** Adult adhd, Work, Stress, Mental health, Qualitative content analysis

## Abstract

**Background:**

Though many adults with ADHD underperform professionally, are more stressed, and have more days of sickness absence compared to adults without ADHD, few studies have explored the experience of working as an adult with ADHD. This study explores the general experience of working with ADHD, including stress and work-related mental illness.

**Methods:**

Semi-structured telephone interviews were conducted with 20 working adults with ADHD. Interview topics included how the ADHD diagnosis and/or symptoms of ADHD may have affected participants on the job, how work may have affected participants’ well-being, and the need for support and accommodation. Qualitative content analysis was used to explore verbatim transcripts from the interviews.

**Results:**

The analysis yielded three themes that describe some of the challenges of working with ADHD: *Working and living with ADHD*, *Needs*, and *Special abilities*, with a total of eight subcategories. Subcategories were *Specific challenges*; *Relationships and cooperation*; *Negative consequences*; *Planning, prioritization, organization, and structure*; *Support, interventions, accommodations, and aids*; *Openness, understanding, and acceptance*; *Strategies*; *Strengths and qualities*.

**Conclusion:**

Further knowledge about the challenges of working with ADHD is needed in workplaces; where organizational support is lacking, much in terms of accommodations and aids is up to the employee, and the disclosure of diagnoses may be associated with great dilemma.

**Supplementary Information:**

The online version contains supplementary material available at 10.1186/s12888-022-04409-w.

## Background

During the last two decades, the proportion of sick leave in Sweden due to psychiatric diagnoses has increased drastically. In 2016, psychiatric diagnoses accounted for 27% of newly registered and 44% of ongoing sickness cases [1]. In Sweden, psychiatric diagnoses are associated with the longest periods of sick leave, longer than for cardiovascular disease and cancer [2]. Similar trends have been observed in many OECD countries, at least since the mid-1990s [3]. The increase in sick leave due to psychiatric diagnoses has been attributed to several factors, including changes in working life, health care, and other aspects of everyday life. Lidwall et al. [4] point to a transition from physical to psychosocial strain, while Lidwall and Voss [5] point specifically to difficulties balancing full-time employment and family obligations.

Adults with attention deficit hyperactivity disorder (ADHD) may have particular difficulties balancing work, leisure, and family, and run a high risk of suffering from mental illness. ADHD is characterized by symptoms of inattention, hyperactivity, and impulsivity. It is one of the most common psychiatric disorders among children and adolescents, and for most patients, symptoms persist throughout adulthood, with associated negative outcomes on both individual and societal levels [6]. Worldwide estimated prevalence of adult ADHD varies between studies, depending on population, methods, and diagnostic criteria, but is estimated to be between two and five percent (e.g., [7, 8]).

Many adults with ADHD underperform academically and professionally, compared to their intellectual potential [9]. ADHD adults report significantly higher work impairment than controls regarding performance and effectiveness as well as attendance, teamwork, and interaction with supervisors [10]. According to Adamou et al. [11], while adults with ADHD may be highly motivated employees, ADHD-related difficulties will soon begin to adversely affect work performance. Some manage to compensate with administrative support or modifications to the working environment, others with well-chosen careers such as creative professions or elite sports, but symptoms of inattention, hyperactivity, and impulsivity are limiting in all but some workplaces. Many adults with ADHD have more difficulty than others managing time, organizing work, prioritizing tasks, following instructions, and regulating emotions. While some can hyperfocus on interesting tasks, this may increase the risk for workaholism.

The relationship between workaholism and ADHD has been demonstrated by Andreassen et al. [12], among others. In addition to the ability to hyperfocus, Andreassen et al. also suggest a compensation hypothesis where adults with ADHD may spend evenings and weekends working to perform on par with their colleagues or to work without distractions. Furthermore, Andreassen et al. suggest that the inattentive nature of ADHD may lead to procrastination and perfectionism, while the impulsive nature may cause individuals to take on more tasks than they can handle. This may be aggravated by hyperactivity, which, combined with difficulties relaxing, may lead to stress and occupational burnout.

The relationship between symptoms of ADHD and stress has been demonstrated by Combs et al. [13], among others. They found inattention, rather than hyperactivity, to be the most consistent predictor. Rogers et al. [14] demonstrated that adults with ADHD were more fatigued than controls, while Brattberg [15] showed a strong association between ADHD, burnout, and long-term sick leave. In a large population-based study of twins, Friedrichs et al. [16] found that symptoms of ADHD were associated with an increased risk for stressful life events, such as divorce, loss of a job, or financial loss. As previously mentioned, adults with ADHD also run a high risk of suffering from mental illness. Several studies have found that most adults with ADHD meet the criteria for at least one comorbid psychiatric diagnosis (e.g., [7, 17, 18].

Researchers studying the economic burden of adult ADHD point to increased costs for sick leave [19]. De Graaf et al. [20] found that workers with ADHD had an average of 8.4 excess sickness absence days per year, compared to otherwise similar respondents without ADHD. In a sibling comparison cost analysis, Daley et al. [21] found that adults with ADHD had an average of 34 sickness absence days per year, compared to an 8-day average among their siblings without ADHD. In a study on data from a large manufacturing firm, Kessler et al. [22] found that 20% of workers with ADHD had one or more sickness absence days in the past 30 days, compared to 10% of workers without ADHD.

In a study of ADHD outpatients aged 19 to 29 years, 38% received sickness absence recommendation, compared to 2.4% in the general population receiving that benefit [23]. All patients included in the study received specialized treatment for ADHD, yet many had a significantly impaired ability to work.

In Sweden, the position of professionals with ADHD in the labor market has been emphasized by government agencies (e.g., [24]), patient interest groups (e.g., [25]), and clinicians. Internationally, similar calls of attention have been made by researchers (e.g., [11]), global professional associations (e.g., [26]), and non-profit organizations (e.g., [27]). Even though many adults with ADHD underperform professionally, are more stressed, have more days of sickness absence, and more often receive sickness absence recommendation, few studies have explored the experience of working as an adult with ADHD.

In one of the most comprehensive qualitative studies to date, Brod, Pohlman, et al. [28] conducted focus groups and interviews to study the general burden of illness for adults with ADHD. Work and career were important topics in all focus groups, with work requiring both coping skills and support. Among the identified obstacles at work were disorganization, inattention, and problems with authority.

Interviewing adults with ADHD between the ages of 21 and 38, Ek and Isaksson [29] found that work can be supportive, through structure and demands, if adapted to individual capacities. Participants appreciated rules and limits for what activities to perform at work, facilitating focus and realization. Participants also described how employment gave value to their lives and provided a social context. Similar results were found in a study by Goffer et al. [30]. In a sample of students, where many combined studies with working, participants described how work provided relatedness, a sense of belonging, and value beyond mere monetary compensation.

Studying experiences of young adults with ADHD and the role of context, Lasky et al. [31] report that for some, stressful and challenging work may alleviate symptoms, by forcing attention and keeping boredom at bay. Interviewing successful adults with ADHD specifically, Sedgwick et al. [32] identified several positive aspects of ADHD. While many aspects were relevant to people in general, a few were regarded as ADHD specific, including *divergent thinking*, *hyper-focus*, and *nonconformist*.

Interviewing a well-educated, high-income sample of older adults with ADHD, Brod, Schmitt, et al. [33] report that participants described accumulated negative ADHD effects on educational achievements, job performance, and subsequent financial status. Participants also described how the stress of ADHD exacerbated other stressors in their lives, with an aggregated negative effect on quality of life. Interviewing older adults with previously undiagnosed ADHD, Michielsen et al. [34] found participants to be hard workers, with some working too hard due to difficulties saying “no”. Some specifically reported overstepping their physical boundaries, culminating in physical ailments due to stress.

Quantitative studies of occupational issues among adults with ADHD tell us much about the negative consequences of working with ADHD. While previous qualitative studies provide further insight into the experiences of working with ADHD, more research is needed on how adults with ADHD experience their working life. This includes specific occupational challenges, successful strategies for coping, and the relationship between work and mental health. This project aims to further study the general experience of working with ADHD, specifically explore stress and work-related mental illness among adults with ADHD, and identify needs in preventing these negative outcomes.

## Methods

In-depth interviews with 20 adults with ADHD were conducted between May and July 2021.

### Participants

Participants were recruited primarily in collaboration with a large Swedish ADHD patient interest group, Riksförbundet Attention. Information about the study was published on social media, together with a link to a website where interested individuals were invited to read more about the study. Terms and conditions were detailed on the website. Before recruitment, the study received ethics approval from the Swedish Ethical Review Authority (Diary number: 2021–01029). Participants were required to be 20–64 years old (Swedish definition of working age), have a diagnosis of ADHD, speak Swedish, work half-time or more, and have work experience equivalent to at least three years full-time. No compensation was awarded for participation. Provision of informed consent was required to submit a notice of interest, thus, written informed consent was obtained from all participants. With this notice, data were collected regarding age, gender, education level, occupational status, work experience, and ADHD diagnosis. Finally, participants submitted their email addresses. In a few days, 90 notices of interest were submitted, and the website was closed to new submissions. All participants were given a random identification code (e.g., 1a2b), and their contact information was stored separately from all other data. Thus, participation was pseudonymous.

### Sampling

From the pool of notices of interests, 20 individuals were chosen purposefully and stratified to reach a heterogeneous sample concerning demographics. Half identified as women, half as men. Ages ranged from 23 to 60 years, education level ranged from high-school degrees to more than three years of university studies, and work experience ranged from 5 to 40 years. These individuals were contacted via email and invited to schedule an interview. If unavailable, or not responding, another individual of similar age and, if possible, the same gender was contacted. In total, 61 individuals were contacted before 20 interviews had been scheduled and conducted. Table 1 shows the characteristics of the final sample. As few men submitted notices of interest, the final sample was predominantly female.

**Table 1:** Participants’ Characteristics

	Participants (*n* = 20)
Age (years): *M (SD)*	41.9 (8.61)
Gender: *n* (%)	
Male	5 (25)
Female	15 (75)
Education level: *n (%)*	
Elementary school	0 (0)
High school	3 (15)
University ≤ 3 years	5 (25)
University > 3 years	12 (10)
Work experience (years): *M (SD)*	17 (8.94)

### Data collection

Semi-structured interviews were conducted via telephone by authors M.O. and M.N. The interviews were recorded using digital audio recorders. All recordings were transcribed verbatim by the interviewers. Identifying information was substituted with generic analogs, e.g., “employer”, “city”, or “care provider”.

An interview guide was used through all interviews (see Additional file 1). The guide was developed by the authors, based on previous research and topics relevant to the research aims. In the first part of the interview, topics included experiences of current and previous workplaces and jobs, how the ADHD diagnosis and/or symptoms of ADHD affected participants on the job, and experiences of support from colleagues, managers, family, friends, and professionals, including accommodations and resources in and around the workplace. In the second part of the interview, topics included how work may have affected participants’ well-being, how the ADHD diagnosis and/or symptoms of ADHD may have influenced that relationship, and the current need for support and accommodation in and around the workplace. Finally, in preparation for an intended future study, participants were asked about their interest in, and attitudes toward, an internet-based psychological intervention targeting stress and work-related mental illness among professionals with ADHD.

### Analysis

A conventional approach to qualitative content analysis was used to explore the content of the interviews [35]. The analysis was conducted by M.O. and M.N., under the supervision of A.R. Both M.O. and M.N. are licensed clinical psychologists, trained and experienced in the assessment and treatment of adult ADHD.

First, all transcripts were read to the letter. Quotes relevant to the research aims were highlighted and extracted, i.e., all quotes regarding work and explicit, implicit, or possible mental illness, including stress and potential stressors. Quotes regarding the intended future study were considered and included if the content also concerned the current research aims. The selection of quotes was continuously discussed between M.O. and M.N., with assistance from A.R. All quotes were then condensed into units of meaning, using the original wording of the participants. Throughout this process, some quotes were shortened, and some excluded, with respect to the research aims. Again, this included a continuous discussion between M.O. and M.N., with regular consultation of A.R. Finally, all units of meaning were coded inductively, as true as possible to the original wording of the participants.

Next, all codes from one interview were extracted, organized, and categorized by M.O. and M.N., individually. Similarities and differences in the results were discussed until consensus was reached. The final categories were compiled in a mind map, with their respective codes. Then, the remaining material was divided between M.O. and M.N. Codes from all interviews were separately extracted, organized, and categorized into mind maps. The result of each categorization was thoroughly reviewed in discussion between the coders until consensus was reached. Finally, the results from all interviews were reviewed collectively, to develop concluding themes. Categories from all interviews were gradually clustered, with close attention paid to maintaining internal homogeneity and external heterogeneity between the final themes [36].

## Results

**Fig. 1:**
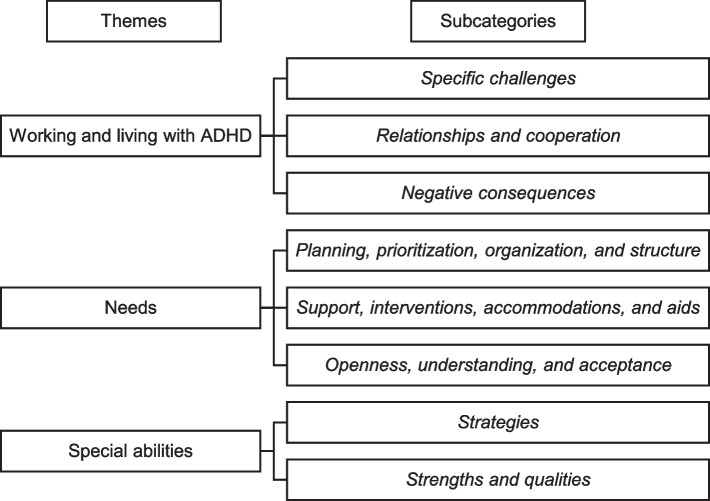
Themes and Subcategories in Data

As presented in Fig. 1, the analysis yielded three themes that describe some of the challenges of working with ADHD: *Working and living with ADHD*, *Needs*, and *Special abilities*, with a total of eight subcategories.

### Working and living with ADHD

Topics related to working and living with ADHD, naturally, comprised large parts of the interviews. This includes experiences and consequences of working with symptoms of ADHD, before and after diagnosis, in different workplaces, in different stages of life, and the circumstances that may mitigate or aggravate existence.

#### Specific challenges

All participants described specific challenges related to working with ADHD, concerning symptoms and/or situations. Among the prominent symptoms were those commonly associated with ADHD, such as distractibility, forgetfulness, restlessness, impulsivity, and impatience. Also prominent were symptoms less typically associated with ADHD, such as emotional dysregulation. Many participants described a hypersensitivity to criticism and failure, and a general sensitivity to changes in mood and atmosphere in and around the workplace, negatively affecting work performance and psychological well-being:

*I feel like I’m very sensitive to criticism, and to things not going my way. I easily fall into believing I’m drastically inferior to my colleagues, even though I know I’m not. I take everything in, it becomes a strong sensitivity to such things, which can cause a dip in energy, or cause my motivation to drop, or make me feel sad. (Participant A)*

The sample was heterogeneous regarding professions, and, as such, challenging situations varied between participants. Recurring, however, were monotone and repetitive tasks, which caused under-stimulation, boredom, and subsequent dejection. Another typical challenge were meetings. Problems with meetings included difficulties staying focused and keeping up with the discussion, trouble gathering one’s thoughts while conversations derail, and stress building up as meetings go overtime:

*I get really stressed out from knowing we won’t get through the agenda. It makes it hard to focus on anything else than seeing the time run out and knowing we won’t finish. It also makes it impossible for me to keep up with the discussion. I can’t keep up with the discussion while focusing on what I think the meeting should really be about. It’s too many discussions and conversations at the same time, on different topics in different areas. (Participant B)*

#### Relationships and cooperation

A large majority of participants mentioned experiences of working alongside and together with other people, and how those encounters affected their experiences of working with ADHD. Prominent was the sense of other’s, imagined or true, judgment of oneself. Some participants described coming across as lazy, ignorant, or incompetent, when dysexecutive. Others described repeatedly being perceived as too much or too straightforward. Many also recalled a history of faux pas in the workplace, as a result of tactlessness and indiscretion:

*It’s taken a while for my colleagues. They’ve thought I’ve been angry or, well, been in the mood. They feel it’s a little uncomfortable. And I’ve told them what’s what, that I’m saying whatever I’m feeling or thinking, out loud. (Participant C)*

#### Negative consequences

Whether participants enjoyed their job, had a good relationship with colleagues, and felt support from management in their vocational aspirations, they all described indisputable negative consequences of ADHD, related to their work. These consequences included negative effects on family and recreation, loss of jobs, unemployment, and symptoms of mental illness. Most prominent among symptoms of mental illness were anxiety, stress, and exhaustion. Several participants described a working life marked by constant anxiety, with spikes in intensity related to work and life events:

*A feeling of impending danger, unease, but you never know what for. It afflicts everything you’re thinking about in that moment. (Participant D)*

*It’s not like you have anxiety for a moment and then you’re good. It just lays there, like a tone, disturbing. (Participant E)*

Regarding stress, many participants described a general sensitivity to stress and feelings of being easily overwhelmed. This was related to feelings of not doing good enough, not keeping up with tasks, and not staying on top of things. For many, this was mediated by symptoms of inattention, such as distractibility and forgetfulness. Some compensated by being exceedingly thorough or working overtime, further depleting their energy reserve. Several participants described being “weathered” by years of stressful work and repeated relapses of exhaustion.

Just as work affected their mental health, many participants also described how their mental health affected their work, positively and negatively:

*If I’m stressed out, and ashamed, and feel stupid, I perform worse at work, too. The effect is crystal clear. In the moments I’m a little happier or more positive, I also perform better. (Participant B)*

#### Other aggravating circumstances

Many participants described circumstances outside work that aggravated the challenges of working with ADHD. Most pronounced were trials related to family and parenthood. Several participants mentioned having children with special needs of their own, and the challenges of working while accommodating them. Not knowing if your child will be able to get out of bed in the morning when you have an important meeting, managing appointments with pediatricians, and always being on-call if something happens at school, introduces another level of strain. A number of participants described the work-family balance as “unmanageable”, fighting to uphold a low-arousal approach when returning home from work, battered.

### Needs

In contrast to specific challenges, negative consequences, and aggravating circumstances, all participants shared thoughts about their current needs, as well as their experiences of successful and unsuccessful interventions, accommodations, and collegial/managerial support.

#### Planning, prioritization, organization, and structure

Many participants experienced great deficiencies in organization and structure at their workplaces, encumbering not only themselves and fellow coworkers with ADHD, but most, if not all, employees. Some described lacking job descriptions and not knowing what to do, leading to a loss of time and energy:

*All the little things like deciding for yourself in what order things should be done, how they should be done, what exactly we are in need of… Those things take a lot of time and energy for me, which it doesn’t always do for my colleagues. (Participant F)*

Participants with experiences of working with clear-cut job descriptions, explicit routines, and consistent implementation, on the other hand, recall thriving and outperforming colleagues. For some, the benefits of structure at work carried over to personal life:

*The structure of having a job helps me out a lot in my spare time. I never take more than ten days off from work, because I don’t want to. I need the trot of working, to maintain any kind of structure in my life. (Participant D)*

#### Support, interventions, accommodations, and aids

Experiences of managerial support varied greatly between interviews. While many participants described supervisors and managers as being supportive, the responsibility for designing and implementing accommodations was often appointed the individual:

*The answer is always “what can you do to make it better?”. I feel like I’m doing everything I can. (Participant B)*

While the experiences of managerial support varied, many participants described supervisors and managers as important or crucial in their experience of working. Certain weight was given to the general attitude of superiors regarding support, and several participants described how a perceived lack of responsiveness could be considerably discouraging. Some participants had greater success working with colleagues to adjust routines and processes in the workplace. For example, some participants had agreements with team members regarding the distribution of tasks. Others found great support in colleagues with similar or other special needs of their own.

Although a small number of participants used assistive technology, such as visual timers and screen readers, very few had been in contact with an occupational therapist. Most participants, however, used mobile and computer applications to organize, plan, and prioritize their work, including intelligent to-do lists, reminders, calendars, and timers. In all cases, the selection and implementation of such applications were done by participants themselves, without professional assistance:

*I have a very detailed to-do list on my computer. I move things around on the list, rather than checking them off, so that I can see I’ve made progress. … The way I’ve set that up, I’ve come up with myself. (Participant E)*

Regarding healthcare support, many participants described significant improvements in functioning at work following pharmacological treatment of ADHD symptoms. Experiences of other interventions in routine psychiatric care, however, were scarce, and generally disappointing. For example, some participants recalled experiencing a lack of knowledge among healthcare providers, beyond basic diagnostic criteria for ADHD. Several participants desired interventions catered to relatively functional adults, focused more on executive function rather than basic organization skills.

#### Openness, understanding, and acceptance

Many participants described how coming to terms with their ADHD-related deficits, often through diagnosis and subsequent (self-)education, enabled them to work constructively with their challenges at work. For some, it also facilitated self-compassion. Experiences of disclosing the diagnosis at work, however, varied. Some shared it confidentially with select colleagues, some only with their closest supervisor, and some spoke openly about it. Other participants were vocal about specific deficits, symptoms, and challenges, rather than disclosing their diagnosis:

*I’ve gotten used to being open with my needs, both with my colleagues and with my boss. I usually don’t talk about my diagnosis, but I wouldn’t lie if someone asked. However, I do feel that I have to be open with the fact that I need help organizing things. (Participant G)*

Responses to disclosure also varied. Some participants experienced significant relief and improved relationships with coworkers. Others recall supervisors and managers not knowing how to deal with the information, and a general lack of understanding of the challenges of adult ADHD. Many participants described a dilemma surrounding disclosure. On the one hand, disclosure may facilitate accommodation, while raising awareness. On the other hand, a diagnosis should not be a prerequisite for accommodation, and the actual informational value of a diagnosis is limited. Several participants also feared discrimination or bias.

### Special abilities

While there was a clear consensus among participants that advantages of ADHD symptoms or an ADHD diagnosis were slim to none, nearly all participants described acquired strategies, personal strengths, and unique qualities that significantly improved function and sometimes alleviated burden at work.

#### Strategies

Many participants employed formal analysis and rigorous problem solving to understand and deal with challenging situations and circumstances at work. Some described performing it on the spot when faced with a challenge, while others made notes of situations during the day to later analyze and try to remedy:

*During a day, I often make lists of things I feel discontent with. That way, I have them recorded to return to, instead of carrying them with me and feeling it’s something personal. You have to maintain a more pragmatic view of things. (Participant H)*

Together, the participants described a wealth of solutions to challenges at work. Beyond positive effects of the beforementioned analyses, efficient approaches included stimulus control, frequent breaks, and intentional gaps in the agenda. Many participants also mentioned successful preventive strategies, including engagement in exercise, outdoor life, and other valuable pastimes.

#### Strengths and qualities

Many participants described a certain creativity and quick-wittedness. This would be expressed in situations such as brainstorming meetings and problem solving, as well as in social settings. For some participants, these traits would go hand in hand with a perceived above-average ability to think outside the box and come up with effective solutions when colleagues and partners were stuck:

*I find it easy to come up with multiple ways to solve a problem, and I’m able to choose between them. Also, I never get stumped in a situation, whereas I often see colleagues get completely paralyzed. (Participant E)*

However, many participants described their wealth of ideas as both a blessing and a curse, as concurrent impulsivity could lead one to express ideas, including possibly controversial ones, in situations where one would be better off keeping quiet:

*I have a huge number of ideas of things to do. I’ve learned to keep quiet about that, sometimes, because you always end up having to do them yourself. (Participant E)*

Some participants described a positive ability to hyperfocus, being able to direct all their attention to the task, activity, or client at hand, or being able to quickly assimilate crucial information on a topic. A few participants also described certain positive effects of hypersensitivity, such as being able to instantly pick up on changes in mood, attitude, or atmosphere in important meetings, and being able to better empathize with clients.

## Discussion

This study explored self-reported ADHD-specific experiences of working with ADHD, including work-related stress and mental illness. Qualitative content analysis of transcripts from 20 interviews revealed three themes of common experiences. The results expand our knowledge on how ADHD adults experience their working life, including specific occupational challenges, experiences of previous interventions, current needs, special abilities, and strategies for coping.

Regarding living and working with ADHD, all participants described specific challenges concerning certain symptoms and situations. In addition to the familiar ADHD symptoms of inattention, hyperactivity, and impulsivity, many participants described a prominent burden of emotional dysregulation, as well as general social hypersensitivity. While current diagnostic criteria for ADHD does not include emotional dysregulation as a core symptom, it has been extensively discussed in the literature whether problems with emotional regulation should be considered a primary symptom of (adult) ADHD (e.g., [37–39]). Barkley and Fischer [40] argue that emotional impulsiveness is as much part of ADHD as inattention, hyperactivity, and impulsivity, with a unique contribution to impairment. Barkley and Fischer also demonstrate associations between emotional impulsiveness and several measures of occupational outcome, beyond the associations of traditional ADHD symptoms. Social sensitivity was also prominent in the results of Sedgwick et al. [32], though discussed not only as a burden but as a complex phenomenon characterized, at its best, by enthusiasm and energy. The clear incidence in current data of issues and impairment related to emotional dysregulation and general social hypersensitivity further emphasize the need for future consideration of these factors in studies on the assessment and treatment of adult ADHD.

Concerning the consequences of living and working with ADHD, several participants described careers characterized by persistent anxiety, stress, and relapses of exhaustion. As previously shown, the association between ADHD, stress, and mental illness, is well documented in quantitative studies. The results of the current study expand our understanding of this relationship, identifying feelings of being overwhelmed, not doing good enough, not keeping up with tasks, and not staying on top of things, as related to perceived stress and overload.

Regarding interventions, accommodations, and collegial support, many participants described negative effects of inadequate organization and structure at their workplaces, while some described thriving under better managerial circumstances. The importance of clarity in communication between managers and employees with ADHD has been emphasized by ADHD experts (e.g., [41]) and management scholars (e.g., [42]) alike. These results are also reminiscent of the support through demands and structure described by Ek and Isaksson [29]. The results of the current study further support the notion that managerial and organizational factors play a vital role in the well-being and prosperity of professionals with ADHD.

While many participants described a significant improvement in working ability after pharmacological treatment of ADHD symptoms, experiences of any further support in healthcare were few and unsatisfactory. While the effectiveness of medications for adult ADHD is well documented (e.g., [43]), national and international guidelines consistently advise multimodal and multidisciplinary treatment (e.g., [44–47]). The results from this study indicate deficiencies in both the provision and the acceptability of non-pharmacological treatment for adult ADHD, among working adults. This includes occupational therapy, as very few participants in the current study had ever been in contact with an occupational therapist, despite consensus among ADHD experts regarding the importance of occupational therapists in ADHD interventions [48].

For many participants, circumstances outside work, including family and parental obligations, aggravated the challenges of working with ADHD. However, nearly all participants also described strategies, strengths, and qualities that improved function and alleviated burden. For many participants, formal analyses and problem solving, combined with creativity and quick-wittedness, was a recipe for progress at work. While planful problem solving is a challenge for some with ADHD, these results are in line with the adult-ADHD tendency to positively reappraise current situations and “continually assess, re-assess, compensate and adapt” ([49] p.814). The merit of creativity and quick-wittedness is also in line with the divergent thinking described by Sedgwick et al. [32].

Qualitative content analysis was used to explore the content of the interviews. The interviews were conducted and transcribed by the authors who later performed the analysis, making for great familiarity with data and the manifest content [50]. For trustworthiness, the analysis process has been described in detail [51], facilitating the readers’ evaluation of its transferability [52]. For credibility and dependability, all parts of the analysis were continuously discussed between the authors, and representative quotes from the transcripts are presented to illustrate how categories and themes cover data [52, 53]. In addition, the process of recruitment and sampling has been described, and the interview guide has been made available.

In all qualitative research, researchers must take into consideration their *pre-understanding*, i.e., their knowledge of the subject and their familiarity with the context [36]. In this study, the authors responsible for conducting the interviews and analyzing data were clinical psychologists experienced in the assessment and treatment of adult ADHD, both having met many ADHD patients struggling with stress and work-related mental illness. Pre-understandings from this clinical work may imply a risk for over-interpretation and confirmation bias regarding the relationship between ADHD (symptoms) and work, and the relationship between work and mental illness. However, it may also guide the evaluation and linking of data, including the appraisal of the novelty and relevance of the findings [54]. In the current analysis, great care was also taken to use and stay true to the original wording of the participants in condensation and coding, i.e., not reading or looking for something between the lines.

The limitations of the current study include the recruitment and sampling process. In the information about the study, the aim of studying work-related stress and mental illness was explicit. It is possible that this framing attracted certain interest from adults with ADHD and a more extensive history of stress and work-related mental illness. While this was of interest in the study, perhaps the sample would have been more heterogeneous had the initial information been less specific. The sample did, however, have an appropriate range regarding age, work experience, education level, and profession.

Future studies may well consider ethnographic methods to further study the challenges of working with ADHD. For example, researchers may consider observing adults with ADHD in the working environment, to gain further knowledge of specific settings and situations that prove challenging. Future research could also focus on the managerial partners in organizations, e.g., experiences of supervising employees with ADHD and managing the challenges and consequences of ADHD in the workplace.

## Conclusions

The results of the current study suggest further knowledge about the challenges of working with ADHD is needed in workplaces; where organizational support is lacking, much in terms of accommodations and aids is up to the employee, and the disclosure of diagnoses may be associated with great dilemma. The results of the current study may also inform other social partners, e.g., labor organizations and work environment authorities, and advise regulations, recommendations, and legislation. More knowledge is also needed in health care, where, other than medications, common interventions in routine psychiatric care seem to generally disappoint working adults with ADHD. While psychoeducational interventions on ADHD in general may be provided, participants in the current study asked for interventions catered to relatively high-functioning patients, and the specific challenges they may be facing.

## Supplementary Information

**Additional file 1.** Interview guide.

## Data Availability

Data from the current study are not publicly available due to them containing information that could compromise research participant privacy. Participants neither consented to data being made available. However, data is archived for at least ten years in compliance with Stockholm University regulations. Requests for any data should be addressed to the corresponding author.

## Abbreviation

ADHD: Attention deficit hyperactivity disorder
