# Supplementary material for: Stress and work-related mental illness among working adults with ADHD: a qualitative study
Source: BMC Psychiatry. 2022 Nov 30;22:751. doi: 10.1186/s12888-022-04409-w (PMC9714234; doi:10.1186/s12888-022-04409-w)
Supplement: Supplementary file 1 — Additional file 1. Interview guide. [file 12888_2022_4409_MOESM1_ESM.pdf]

Interview guide

Background

- ❑ What does your household look like? Do you live with someone or some?
    - Do you have kids?
- ❑ What is your education?
- ❑ Tell me about your diagnosis.
    - How did you get diagnosed?
    - What was that like for you?
    - In what ways has the diagnosis changed your everyday life (e.g., at work)?
    - Do you have any other (neuro-)psychiatric diagnoses?

Work

- ❑ What is your current employment?
    - How long have you been working there?
    - How long have you been working as [profession]?
    - How do you like your current responsibilities?
    - How do you like your current workplace?
    - What is your previous professional experience (e.g., previous jobs)?
- ❑ How do you feel your ADHD/ADHD symptoms affect you at work?
    - In your performance?
    - In the workplace (e.g., with colleagues)?
    - Positive effects/strengths?
    - Negative effects/challenges?
    - How did it affect you previously in your professional career?

- ❑ How do you deal with your challenges at work? [Use any examples from earlier]
    - Is there a basis for support when you need it?
    - Do you receive support from others at work (e.g., colleagues or a manager)?
    - Have you told anyone at work about your ADHD (e.g., colleagues or a manager)? If so, what was that like?
    - Do you use any aids (e.g., cognitive aids)?
    - Do you use any other kind of support, aids, or alike?

Mental health

- ❑ Has your work affected your mental health?
    - For example, have you felt stressed, depressed, or worried?
    - Is that in certain moments or in connection to certain tasks?
    - How do you think that may have affected your everyday life in general?
    - How do you think that, in turn, may have affected your work?
    - Do you see any connection between this and your ADHD/ADHD symptoms?
- ❑ If you have experienced negative effects on your mental health, related to your work, how have you managed that?
    - Support from friends and family?
    - Speaking to your manager or your colleagues?
    - Professional support (e.g., in health care)?

Prevention

- ❑ What are your current needs for support at work?
    - [Concerning previously mentioned challenges and effects]
    - What kind of support do you think would be helpful for you?

- How would you feel about taking part in an internet-based program designed to prevent stress and work-related mental illness among adults with ADHD? For example, it could contain information and exercises relevant to challenges experienced by many professionals with ADHD.
  - What are the possible upsides of such a program?
  - What are the possible downsides of such a program?
- If you were involved in the development of such a program, what would you include?
  - How long would it be?
  - What would be the focus?
  - What is important to keep in mind when developing such a program?
  - Is there something you think must be included?
  - How much time is reasonable for such a program to call upon?
- What things could…
  - …increase the likelihood that you take part in it?
  - …stop you from taking part in it?
  - …make you lose your motivation?
  - …make you quit before the program was finished?
- What do you think about having a psychologist as a therapist in the program, compared to doing it all on your own?
  - What could the advantages be?
  - What could the disadvantages be?
  - If the program would include contact with a psychologist, how often would you like to be in contact with them?
  - Would the contact be scheduled or at your discretion?

Conclusion

- ❑ Is there anything you would like to add?
